# Child and adolescent psychiatric patients and later criminality

**DOI:** 10.1186/1471-2458-7-221

**Published:** 2007-08-29

**Authors:** Ulf Engqvist, Per-Anders Rydelius

**Affiliations:** 1 Department of Women and Child Health, Karolinska Institutet, Astrid Lindgren Children's Hospital at Karolinska University Hospital, SE-171 76 Stockholm, Sweden; 2 Department of Social Work, Mid Sweden University, SE-831 25 Östersund, Sweden; 3 Department of Women and Child Health, Karolinska Institutet, Astrid Lindgren Children's Hospital at Karolinska University Hospital, SE-171 76 Stockholm, Sweden

## Abstract

**Background:**

Sweden has an extensive child and adolescent psychiatric (CAP) research tradition in which longitudinal methods are used to study juvenile delinquency. Up to the 1980s, results from descriptions and follow-ups of cohorts of CAP patients showed that children's behavioural disturbances or disorders and school problems, together with dysfunctional family situations, were the main reasons for families, children, and youth to seek help from CAP units. Such factors were also related to registered criminality and registered alcohol and drug abuse in former CAP patients as adults. This study investigated the risk for patients treated 1975–1990 to be registered as criminals until the end of 2003.

**Methods:**

A regional sample of 1,400 former CAP patients, whose treatment occurred between 1975 and 1990, was followed to 2003, using database-record links to the Register of Persons Convicted of Offences at the National Council for Crime Prevention (NCCP).

**Results:**

Every third CAP patient treated between 1975 and 1990 (every second man and every fifth woman) had entered the Register of Persons Convicted of Offences during the observation period, which is a significantly higher rate than the general population.

**Conclusion:**

Results were compared to published results for CAP patients who were treated between 1953 and 1955 and followed over 20 years. Compared to the group of CAP patients from the 1950s, the results indicate that the risk for boys to enter the register for criminality has doubled and for girls, the risk seems to have increased sevenfold. The reasons for this change are discussed. Although hypothetical and perhaps speculative this higher risk of later criminality may be the result of lack of social control due to (1) rising consumption of alcohol, (2) changes in organisation of child social welfare work, (3) the school system, and (4) CAP methods that were implemented since 1970.

## Background

Using longitudinal methods for studying juvenile delinquency is a long-standing research tradition in Swedish child and adolescent psychiatry (CAP). Based on various scientific paradigms (curative education, genetics, neuropsychiatry, and social psychiatry) and since the 1920s, CAP research results were presented using either prospective or retrospective longitudinal designs, which cover 20–40 years of follow-up periods. Psychopathic boys treated at the Mellansjö-treatment home were followed from 1928 and forward to 1968 [1], delinquent boys treated at the Children's Village Skå and average Stockholm boys were followed over 18 years 1954–1973 [2], adoptive children from deviant homes 1930–1972 [3], children of alcoholics 1958–1978 [4], and teenage alcoholics 1964–1985 [5]. A number of Swedish Government official reports described young law-breakers from the general population and their criminal development 1960–1972 [6-10] (see Table 1).

**Table 1:** A summary of Swedish longitudinal prospective studies of risk groups with relevance to CAP and delinquency

Sample	Academic Discipline from which the study originated	Later Criminality	Later Alcoholism	Follow-up period
**Samples of boys who themselves had shown/been treated for early symptoms of delinquency**				
Psychopathic boys treated at the Mellansjö CAP-treatment home	CAP	41%	28%	1928–1968
Delinquent boys treated at the Children's Village SKÅ	CAP	67%	58%	1954–1973
Young law-breakers from the general population	CAP, Sociology, Psychology, Criminology	39%	46%	1960–1972
Teenage alcoholics	CAP	42%		1964–1985
			58%	1964–1977
**Samples of boys who had not primarily shown early symptoms of delinquency**				
Adopted children with heredity for social problems/alcoholism	CAP	14%	21%	1930–1972
Children of alcoholic Fathers	CAP	42%	35%	1958–1978
**Samples of CAP-patients**				
2164 patients from the Stockholm CAP out-patient-service	CAP	16 % (23% of the boys)	16 % 23% of the boys)	1953–1975
		(3% of the girls)	(3% of the girls)	

Results from the descriptions and follow-ups of cohorts of CAP patients until the 1980s [11,12] showed that children's behavioural disturbances or disorders and school problems (with dysfunctional family situations) were the main reasons for families, children, and youth to seek CAP care. Such factors were also related to registered criminality and registered alcohol and drug abuse in former CAP patients as adults [12].

The earlier findings could be summarized in the following way: In cohorts of CAP patients and in comparison groups followed from the 1950s to the late 1970s, male CAP patients had a higher risk of later registered criminality compared to average boys and adoptive children from disturbed homes [3], but a lower risk of criminality compared to children of alcoholic fathers [4] and children with early established conduct disorders [13]. Female CAP patients demonstrated very low risk of later criminality.

This study investigated the risk for CAP patients treated 1975–1990 to enter the register on criminality. The study's hypothesis was that children, who were identified by CAP units as having behavioural and school problems and dysfunctional families, were at high risk to become criminals.

## Methods

### CAP Units

In Sweden, there are three psychiatric disciplines, general (adult) psychiatry, forensic psychiatry and child and adolescent psychiatry (CAP). The upper age limit for CAP care is 18 years. CAP in Sweden was established during the time period 1915–1935 and became a medical discipline with its own training programme and an independent member organisation of the Swedish Society of Medicine in 1956. The influences came from Central Europe, mainly Austria, Switzerland and Germany. A nation-wide organization for CAP health service was built up from a Parliament decision in 1957–1958, and every County council has today at least one CAP unit focusing on outpatient care.

The number of inpatient beds has been reduced by nearly 90% from 1967 to 2006. There are differences in the CAP organisations and traditions over the world as well as when comparing the European countries and the Nordic countries. This is a matter of concern discussing similarities and differences when CAP patients from different countries are compared.

In the Jämtland County there are one CAP clinic and one general psychiatry clinic under the same health organisation giving service to the whole population. Both clinics are located in the same hospital organisation at Östersund Hospital.

### Study sample

The population consists of all patients born between 1957 and 1976, who were admitted to CAP care in Jämtland County between 1970 and 1990. They terminated their contact between 1975 and 1990. Some of the youngest patients might have been readmitted to CAP care after 1990. The data apply to CAP in- and outpatient care and general psychiatric in- and outpatient care in Jämtland County and general psychiatric inpatient care in the rest of Sweden. Only few cases (8) are missing. In total, 99.4% of all patients were successfully traced in Swedish census records; of these, 12 had emigrated.

Jämtland County is a sparsely populated (127,028 inhabitants in December 2005) area in northern Sweden. The county represents 12% of Sweden's land area but only 1.5% of its population. Östersund (about 58,000 inhabitants), the only town in which psychiatric services are located, is 600 kilometres northwest of Stockholm. The County Council organisation and Östersund hospital are well-suited for this type of study. There is only one CAP clinic and one general psychiatric clinic for all county inhabitants; both clinics are in the same hospital organisation.

### The CAP patient population, in brief

Information about CAP care was available for all 1,420 patients, of which 674 were male and 746 were female (female/male ratio 1.1:1). Their mean age at admission to CAP care was 12.1 (SD. 4.1).

In 2003, 896 of the patients (63%) lived in Jämtland County; the others had moved to other parts of Sweden, and 12 had moved abroad. It was not possible to acquire reliable data on eight persons, and another 38 patients (2.7%) had died.

Data on split families were available for 1,399 patients. 47.5% of the patients lived with either both biological or both adoptive parents at admission to CAP care. In the early 1990s, 75% of all Swedish children lived with both parents and that difference is significant (*P *< 0.001). School problems (behavioural and/or learning problems) were mentioned in the hospital records for 46.5% of 1,187 patients.

Every fifth (20.3%) was given in-ward treatment at the CAP clinic. In Sweden there have been efforts to shorten the duration of inpatient treatment and to promote out-patient care from the 1990s and onward. This means that the duration of hospitalizations were rather long during the period from 1975–1990, with an average of 90 days and the range 1–449 days.

### Data collection

Based on the description of the CAP patient population, a prospective survey was conducted with 1,400 patients (including those who died during the follow up) to examine the number of patients who were later convicted of offences. Authorisation and ethical approval was received from the Ethical Committee of Umeå University and the Ethical Committee of Karolinska Institutet in Stockholm (Um dnr 95-051;Um dnr 99-023; KI Dnr 99–209). The Swedish National Council for Crime prevention also accomplished an ethical trial before supporting us with data. Information about contacts with CAP and general psychiatric care was obtained from hospital records and from the Hospital Discharge register (HDR) of the Swedish National Board of Health (covering the whole nation). A list of all social security numbers (all checked against vital statistics) was sent on two occasions (1999 and 2006) to the National Council for Crime Prevention (NCCP) to search in its database of persons who were convicted of offences.

### Information from hospital records

The study started in 1994–1995 when hospital records at CAP were reassessed according to a protocol (based on previous empirical results from follow-ups of CAP patients) to identify the following variables: gender, age when admitted to CAP care, split family, school problems, inpatient care in the CAP unit, reason for admission, and diagnoses. These variables were used in the statistical analysis as independent variables.

Over the years 1975 – 1996, ICD-8 and ICD-9 were used in Sweden for diagnostic purposes. ICD-10 was introduced in 1997. For this study, all diagnoses given at the time of CAP treatment have been reassessed according to ICD-10 which is described in detail on Who's web page [14].

Depending on the history and assessment, patients visiting the outpatient CAP unit do not always receive a psychiatric diagnosis. For one third of the patients (35%) no diagnosis was given. A psychiatric diagnosis (group F, mental and behavioural disorders) was given to 534 patients (38%). Every fifth patient (23%) received a Z group diagnosis (Factors influencing health status and contact with health services). Investigations of relationship problems within the family usually lead to such a diagnosis. Suicide attempts (X60–X84 Intentional self-harm) constituted 5%. The distribution of diagnoses in groups is shown in Table 2.

**Table 2:** Diagnoses (ICD 10) given to 913 patients at the CAP unit

**Diagnosis group**	Total study population	Males	Females	p-value
	N = 913	N = 395	N = 518	
	Number (%)	Number (%)	Number (%)	
**Mental and behavioural disorders**				
F10-F19 Mental and behavioural disorders due to psychoactive substance use	25 (2.7)	11 (2.8)	14 (2.7)	0.93
F20-F29 Schizophrenia, schizotypal and delusional disorders	20 (2.2)	7 (1.8)	13 (2.5)	0.47
F30-F39 Mood [affective] disorders	31 (3.4)	12 (3.0)	19 (3.7)	0.56
F40-F48 Neurotic, stress-related and somatoform disorders	142 (15.6)	42 (10.6)	100 (19.3)	0.0002
F50-F59 Behavioural syndromes associated with physiological disturbances and physical factors	47 (5.1)	9 (2.3)	38 (7.3)	0.0003
F60-F69 Disorders of adult personality and behaviour	4 (0.4)	1 (0.3)	3 (0.6)	0.80
F70-F79 Mental retardation	27 (3.0)	17 (4.3)	10 (1.9)	0.04
F80-F89 Disorders of psychological development	39 (4.3)	26 (6.6)	13 (2.5)	0.04
F90-F98 Behavioural and emotional disorders with onset usually occurring in childhood and adolescence	198 (21.7)	121 (30.6)	77 (14.9)	< 0.0001
F99 Unspecified mental disorder	1 (0.1)	1 (0.3)	0	0.28
**Diseases of the nervous system**				
G00-G99 Diseases of the nervous system	2 (0.2)	2 (0.5)	0	0.16
**Endocrine, nutritional and metabolic diseases**				
E00-E90 Endocrine, nutritional and metabolic diseases	2 (0.2)	1 (0.3)	1 (0.2)	0.77
**Factors influencing health status and contact with health services**				
Z00-Z99 Factors influencing health status and contact with health services	326 (35.7)	139 (35.2)	187 (36.1)	0.78
**External causes of morbidity and mortality**				
X60-X84 Intentional self-harm	49 (5.4)	6 (1.5)	43 (8.3)	< 0.0001

### Information on crimes

For this presentation, the Swedish statistics on crimes have been used. From the BRÅ, The Swedish National Council for Crime Prevention (NCCP), the authority in Sweden responsible for Crime statistics) the following is cited: "Comparisons between countries that are based on their individual crime statistics require caution since such statistics are produces differently in different countries." Swedish statistics on crimes is presented in detail in several languages on the BRÅ web page [15].

Crimes are normally broken down into various categories or groups. There are at least two ways of doing this: the legal approach and the criminological approach.

With legal categorisation, criminal code crimes are normally grouped on the basis of the code's division into crimes against the person, crimes against property, crimes against the general public and crimes against the state.

Criminologists are more interested in grouping crimes that are a similar type, that have the same causes or that occur under similar circumstances. Some of these criminological concepts have also made their way into everyday language, such as environmental crime, juvenile crime and violent crime. Both grouping procedures are used in this study.

Crimes against property are robbery, larceny, burglary, motor vehicle theft and petty larceny.

Crimes against the person's life and health are also called violent crimes. The group contents murder, manslaughter, assault and sexual crimes.

Drug-related crimes are drunken driving, narcotics and crime against the alcohol law. Reoffending is measured on the basis of the new convictions recorded during the follow up period subsequent to an individual's initial conviction.

### Information from registers

NCCP database search results for each person were displayed in three rows, which indicated if the person (1) was found guilty in a county court; (2) had received a fine issued by a prosecutor; and/or (3) had received a waiver of prosecution issued by a prosecutor [16]. A maximum of 10 previous violations of a law were displayed in the first search. If a person had more than ten convictions, this was noted. Only the main crime was noted. Sometimes up to 19 types of offence are committed at the same time, but here, only the most serious crime could be registered, that is, the "main" crime.

The information for outpatient general psychiatric care as well as inpatient care in Jämtland County was obtained by examination of hospital records and by linkage to the Hospital Discharge register (HDR) of the Swedish national Board of Health while information of inpatient care in the rest of Sweden was obtained by linkage to HDR.

### Statistical methods

The odds ratio (OR) is a way of comparing whether the probability of a certain event is the same for two groups. An odds ratio of 1 implies that the event is equally likely in both groups. An odds ratio greater than one implies that the event is more likely in the first group. An odds ratio less than one imply that the event is less likely in the first group. The odds ratio is presented with 95% confidence interval.

The rate ratio is most suited to study events in a constant domain while the denominator -i.e. the population at risk- is very large. The rate ratio is presented with 95% confidence interval (Wald).

The chi square test was used to analyse differences in categorical variables. For continuous variables, the t-test was used to analyse differences. A significance limit was set at 5%.

Binary logistic regression determined effect of a set of variables on probability of criminality – plus effect of individual variables. Binary logistic regression is a regression application for a dichotomous, dependent variable. The logistic regression model is a nonlinear transformation of the linear regression.

Data were analysed using SPSS for Windows (SPSS Inc), release 12.0

## Results

### Registered criminality

A total of 530 persons (38%) from the study population (N = 1,400), 367 males (26%) and 163 females (12%), every second man (55%) and every fifth woman (22%), were registered for criminality. The male/female ratio was 2.3:1.

This study has 279 inpatients, and 44% appeared in criminal databases.

A total of more than 2,000 different crimes were registered for the 530 former CAP patients. Eighty-one types of crimes were found in the records. Repeated criminality was common. It was possible to compare registered criminality among previous CAP patients to a group of average, Swedish males and females in the same age groups for 1996 and 1997. It was shown that 5,682 per 100,000 of males in the CAP population were convicted of offences while 4,113 per 100,000 in the general population were convicted (*P *< 0.001). Rate ratio 95% CI was 0.61 < 1.38 < 3.14. Corresponding figures for females were 1,851 per 100,000 compared to 741 per 100,000, Rate ratio 0.59 < 2.50 < 10.55 (*P *< 0.001).

A total of 315 (59%) reoffended; 223 patients (42% of those convicted) were reconvicted within three years of the first conviction, and 129 (24% of those convicted) reoffended within one year.

### Most frequent-occurring crimes

A total of 2,365 crimes were registered for the 530 former CAP patients. Table 3 lists the seven most common crimes. They constitute 65% of all crimes. The most serious crimes were robbery (10 cases), rape (6 cases), arson (3 cases), manslaughter (2 cases), and murder (1 case).

**Table 3:** The most frequent-occurring crimes in the CAP patient population

**Type of crime**	**Persons convicted of offences**		**Numbers of convictions of offences**		**Crime per person**	
	**Males**	**Females**	**Males**	**Females**	**Males**	**Females**
Larceny, burglary	158	40	404	99	2.6	2.5
Driving without a license	92	17	177	38	1.9	2.2
Motor vehicle theft	91	13	190	15	2.1	1.2
Petty larceny	50	62	70	99	1.4	1.6
Assault	86	20	134	28	1.6	1.4
Drunk driving	90	22	117	28	1.3	1.3
Narcotics	32	31	61	69	1.9	2.3

A total of 530 persons, 367 males and 163 females, were convicted for a total of 2065 crimes.

### Violent offences

Of the total number of reported crimes in Sweden, crime against the person (primarily violent crime) constitutes 15%. Crime against the person includes, for example, assault and sexual crimes.

One-hundred and forty-three persons (115 male and 28 female) had committed a total of 248 violent crimes, which is 1.7 crimes per person. One-third (31%) of the convicted males (17% of all males) and 17% of the convicted females (4% of all females) had committed violent crimes.

Violent crimes constituted 10% of all crimes in this study. The most frequent violent crime was assault (162 or 7%). Sexual crimes constituted 1%.

Those who committed violent crimes differed from those who committed other crimes regarding gender (*P *= 0.001), split family (*P *= 0.035), behaviour disorders (*P *= 0.009), and schizophrenia (*P *= 0.027). They also had a higher degree of general psychiatric contacts (*P *= 0.002).

The schizophrenic patient group did not commit more crimes than patients with other diagnoses, but they more frequently committed violent crimes.

### Drug-related offences

One hundred and seventy-six persons (125 male and 51 female) had committed 300 crimes related to alcohol and drugs, which is 1.7 crimes per person. Crimes related to alcohol and drugs constituted 13% of all crimes. The most frequent crime was drunk driving (145 or 6% of all crimes), followed by narcotics (130 or 5.5%). Those, who committed crimes related to alcohol and drugs, differed from those who committed other crimes – regarding age at admission to CAP care (> 13 years); (*P *= 0.008) and behaviour disorders (*P *< 0.001). The alcohol-drug criminals also had more frequent general psychiatric contacts (*P *= 0.002) and a higher mortality rate (*P *= 0.004).

### Female versus male criminality

In this study, among the 530 individuals convicted of offences, every third person (31%) was female. The 163 females committed 25% of all the registered crimes. Men were convicted for 4.9 crimes per person (SD 5.87) and women for 3.5 crimes per person (SD 5.04); (*P *= 0.006).

The most typical female crimes were pilfering, theft, burglary, and narcotics.

Significant differences between females convicted of offences versus males convicted of offences, and females convicted of offences versus females not convicted are shown in Table 4.

**Table 4:** Females convicted of offences vs. males convicted of offences, females convicted of offences vs. females not convicted, and males convicted of offences vs. males not convicted

	**Females convicted of offences**			**Males convicted of offences**				
	**N**	**Number**	**Percent**	**N**	**Number**	**Percent**	**p-value**	**Odds Ratio 95% CI**
**First conviction before 18 years of age**	153	53	34.6	346	161	46,5	0.013	1.1 < 1.6 < 2.4
**Reoffending**	154	81	52.6	347	236	68.0	0.001	1.1 < 1.3 < 1.5
**Problems at school**	148	72	48.6	299	198	66,2	< 0.001	1.1 < 1.4 < 1.6
**Age when admitted to CAP, younger than 13 years**	163	57	35.0	367	245	66,8	< 0.001	2.5 < 3.7 < 5.5
**Reason for admission to the CAP unit**								
**Behaviour disorder**	161	36	22.4	363	144	39.7	< 0.001	1.3 < 1.8 < 2.4
**Problems in connection with development or maturation**	161	3	1.9	363	26	7.2	0.014	1.2 < 3.8 < 12.5
**Attempted suicide, suicidal acts, or thoughts**	161	12	7.5	363	7	1.9	0.002	1.6 < 4.1 < 10.6
**Relationship problems**	161	55	34.2	363	61	16.8	< 0.001	1.7 < 2.6 < 4.0
	**Females convicted of offences**			**Females not convicted of offences**				
**Problems at school**	148	72	48.6	498	168	33.7	0.001	1.3 < 1.9 < 2.7
**Split family**	159	106	66.7	562	263	46.8	< 0.001	1.3 < 1.6 < 2.0
**Reason for admission to the CAP unit**								
**Behaviour disorder**	161	36	22.4	565	48	8.5	< 0.001	1.9 < 3.1 < 5.0
**Relationship problems**	161	55	34.2	565	101	17.9	< 0.001	1.6 < 2.4 < 3.5
	**Males convicted of offences**			**Males not convicted of offences**				
**Split family**	360	229	63.6	298	126	42.3	< 0.001	1.3 < 1.6 < 1.9
**Problems at school**	299	198	66.2	225	106	47.1	< 0.001	1.3 < 1.6 < 1.9
**Reason for admission to the CAP unit**								
**Behaviour disorder**	363	144	39.7	300	64	21.3	< 0.001	1.2 < 1.3 < 1.4

Percentage is valid for convicted or not convicted females respectively convicted or not convicted males

### Juvenile criminality

Thirty persons were convicted before their first admission to CAP care. Those who were under age 18 at their first conviction constituted 40% of all those convicted, and they were responsible for 59% of all crimes. The 140 patients who were ages 15–16 (at their first conviction) constituted 26% of all those convicted, and they were responsible for 42% of all crimes. Table 5 shows the time from CAP care to first conviction.

**Table 5:** Time from completion of CAP-care to first conviction

Age at first conviction	Frequency	Convicted before CAP-care	Convicted during CAP-care	Time to first conviction after completion of CAP care						
				0–1 year	1–2 years	2–5 years	5–10 years	10–15 years	15–20 years	20–25 years
15	31	3	7	8	2	8	3			
16	109	16	28	18	7	20	18	2		
17	74	11	16	8	13	13	11	2		
18	58		4	12	8	19	10	5		
19	46				3	21	17	5		
20	38				1	13	14	10		
21	37		1			10	17	9		
22	28		1		2	4	12	9		
23	17					2	4	10	1	
24	15						8	6	1	
25	10						4	5	1	
26	10						1	9		
27	3							2		1
28	9							8		1
29	6						1	5		
30	6							6		
31	6							6		
32	3							3		
33	6							5		1
34	7							5		2
35	3							3		
36	1									1
37	2							1		1
39	3									3
40	2									2

Total	530	30	57	46	36	110	120	116	3	12

Those who were convicted five times or more made their criminal debut earlier than others. The mean age for the first crime was 17.4 (SD 2.08), while the corresponding figure for those who committed less than five crimes was age 21.1 (SD 5.46); (*P *< 0.001). Other significant differences (compared to those who committed less than five crimes) were that they reoffended more often (*P *< 0.001), they had more school problems (*P *< 0.001), they were older at first admission to CAP care (*P *= 0.029), and they were more often admitted to CAP care because of behaviour problems (*P *< 0.001). More of them had also been inpatients in CAP care (*P *< 0.001) and had been admitted to general psychiatry (*P *< 0.001).

### Criminality and admission to general (adult) psychiatry

In the CAP patient population, a total of 531 patients (38%) had been patients in general psychiatry care at the end of the follow-up, and among those convicted of offences, 44% had been readmitted to general psychiatric care (*P *< 0.001).

In childhood, during CAP care, those convicted of offences were admitted more often because of behavioural disorder (180/524; 34%) than hose not convicted (112/865; 13%) (*P *< 0.001) and relationship problems (116/524; 22% vs. 142/865; 16%), (*P *= 0.008).

As adults, they had a significantly higher rate of substance-related diagnoses (51/241; 21%) than those not convicted (7/297; 2%), (*P *< 0.001) and personality disorder diagnoses (31/241; 13% vs. 19/297; 6%), (*P *= 0.010).

Those convicted of offences had a significantly lower percentage of schizophrenia (12/241; 5% vs. 29/297; 10%), (*P *= 0.038); neurotic, stress-related, and somatoform disorders (42/241; 17% vs. 90/297; 30%), (*P *= 0.001); and behavioural syndromes that are associated with physiological disturbances and physical factors (16/241; 7% vs. 37/297; 13%), (*P *= 0.024).

### Risk factors

Table 6 displays risk factors for conviction of offences (Odds ratio and a binary logistic regression).

**Table 6:** Risk factors for conviction of offences: Odds ratios and binary logistic regression (forward LR) with 95% CI

**Independent variable**	**N**	**Dependent variable**		**P value**	**Odds ratio**
		Convicted of offences	No conviction of offences		
Sex	1400				
Male	667	367	300		
Female	733	164	569		1.8 < 2.1 < 2.4
*Binary logistic regression*	*1161*			*< 0.001*	*0.2 < 0.3 < 0.4*
Family	1379				
Split family	724	336	388	< 0.001	1.4 < 1.6 < 1.8
Complete family	655	184	471		
*Binary logistic regression*				*< 0.001*	*0.4 < 0.5 < 0.6*
Problems at school	1170				
Problems	544	271	273	< 0.001	1.5 < 1.8 < 2.0
No problems	626	177	449		
*Binary logistic regression*				*< 0.001*	*1.4 < 1.9 < 2.5*
Reason for admission to the CAP unit	1389				
Behavioural disorder	291	179	112	< 0.001	2.7 < 3.5 < 4.5
Other cause for admission	1098	346	752		
*Binary logistic regression*				*< 0.001*	*2.1 < 3.0 < 4.2*
Relationship problems	258	117	141	0.006	1.1 < 1.5 < 1.9
Other cause for admission	1131	408	723		
*Binary logistic regression*				*< 0.001*	*1.5 < 2.1 < 3.0*

P-value and odds ratio from chi-square tests, valid for each variable separately, is shown in plain text. The binary logistic regression odds ratios and p-values are shown in Italic in connection to each variable. See also statistical methods.

## Discussion

### A comparison with a 20-year prospective follow up study in Stockholm and other studies

Nylander [12] published a 20-year prospective follow-up study of 2,164 patients (1417 males and 747 females) from the Stockholm Municipal Child Guidance Clinics. After treatment they had been discharged in 1953, 1954, and 1955.

To compare the Jämtland cohort to the Stockholm cohort, the sub-sample of the Jämtland cohort with a full 20-year follow-up period was selected. This group, 608 cases (325 males and 283 females) was compared to the Stockholm group using the variables described in Table 7.

**Table 7:** A comparison between 2164 CAP patients from Stockholm in the 1950s and 608 patients from CAP in Jämtland using an observation time of 20 years

	**Stockholm**		**Jämtland**		
	Number	Percent	Number	Percent	P-value
Primary material	2364		1420		
Emigrated	100	4.2	12	0.8	< 0.001
Deceased during follow up	50	2.1	38	2.7	0.281
Unusable data	50	2.1	8	0.6	< 0.001
Inpatient care at CAP	0	0	270	19	< 0.001
Less than 20 year follow up	0	0	484	34.1	< 0.001

20 year observation time	2164	91.5	608	42.8	0.004
Males	1417	65.5	325	53.5	< 0.001
Females	747	34.5	283	46.5	< 0.001
Age at end of follow up period					
20–31.5 years	1415	65.4	236	38.8	< 0.001
31.6 years or above	749	34.6	372	61.2	< 0.001
Registered for offences	344	15.9	228	37.5	< 0.001
Males	319	22.5	174	53.5	< 0.001
Females	25	3.3	54	19.1	< 0.001
Males/Females	12.8:1		3.2:1		< 0.001
Mean age at registration					
Males	20.6		19.8		< 0.001
Females	23.0		21.3		0.009
Number of convictions	988		863		
Males	947		681		
Females	41		182		
Males/Females	23.1:1		3.7:1		< 0.001
Convictions per person	2.9		3.8		0.401
Males	3.0		3.9		0.297
Females	1.6		3.4		0.986
Serious violent crimes	15	1.5	11	1.3	0.655

Serious violent crimes are: Murder, manslaughter, attempted manslaughter, cause of another's death, arson, robbery and rape.

Proportions has been compared using t-test (Fisher exact)

Stockholm cohort: Patients that attended the Child Guidance Clinics 1953–55, followed up for 20 years

Jämtland cohort: Patients born 1957 to 1976 whose treatment occurred between 1975 and 1990, followed to 2003, those with less than 20 year follow up excluded

While the boy/girl ratio was 1.9:1 in the Stockholm cohort from the 1950s, more girls were CAP patients in Jämtland at the end of the 1900s – with a 1:1.1 boy/girl ratio.

Compared to 22.5% of the Stockholm boys, 53.5% of the Jämtland boys were registered for criminality over the coming 20 years after discharge from CAP care (*P *< 0.001). Among the girls, the corresponding figures were 3.3% compared to 19.1% (*P *< 0.001). The Jämtland girls were younger than the Stockholm girls at the time of first conviction (*P *< 0.001); while no age difference was found among boys (Table 7).

The Stockholm organisation for CAP outpatient care was established in 1933 and worked hand in hand with the CAP unit on school psychiatry that was established in the Stockholm Public Schools in 1919. As child guidance and prevention was the primary goals for the CAP activities, special attention focused "on the important pre-school years" with the "setting up of special small day care centres with a programme of therapeutic activities connected with the guidance clinics, and special centres for children with grave mental disturbances that also run within the framework of the organisation" (page 11, Curman H., Nylander I. [11]). However, there has been a change observed over time in age and sex distribution of CAP patients in Stockholm over time. "Whereas the frequency for children in the lowest age group during the years 1953–1954–1955 amounted to 24% of the total material, this figure was only 8% (p < 0.001) in 1970; while the frequency for children in the oldest age groups in 1953–1954–1955 was about 12%, it reached 39% in 1970 (*P *< 0.001). The largest increase was found for the girls in the highest age group, rising from about 17% to about 49% (*P *< 0.001)" (p26, Curman H., Nylander I. [11]).

As the fully established organisation in Jämtland started in 1975 working according to the current opinions of the period of time, this may well explain why the proportion of preschool children was higher in the Stockholm cohort in the 1950's and why the Jämtland cohort was older at the end of a 20-year follow up (see Table 7). However, as 92% of the convictions among the Stockholm boys occurred between their ages of 15–26 years vs. 91% in the Jämtland cohort and as there was no age difference found when comparing the two cohorts of males at the time for their first convictions the age difference between the two cohorts is looked upon as negligible.

The sex-ratio where boys outnumber girls, found in the Stockholm cohort from the 1950's, is in line with other CAP-cohorts from that time. A similar situation was found in the description of the CAP patients at the outpatient clinic of the Erica Foundation published in 1950 [17]. As already commented upon above, there was a secular trend observed from 1950–1970 in Stockholm in relation to the sex ratio of CAP patients, when the number of girls increased. In a Stockholm CAP cohort from 1981, the sex ratio girls: boys was 1:1, while in a Stockholm cohort of CAP emergency patients from 1995 the girls outnumbered the boys 60% vs. 40%. This secular trend and the fact that "problematic girls were uncommon among both CAP patients and delinquent children and youth in cohorts in the 1950's" (p3. [18]) may well explain the sex differences found when comparing the Stockholm cohort from 1950 to the Jämtland cohort.

**Figure 1:**
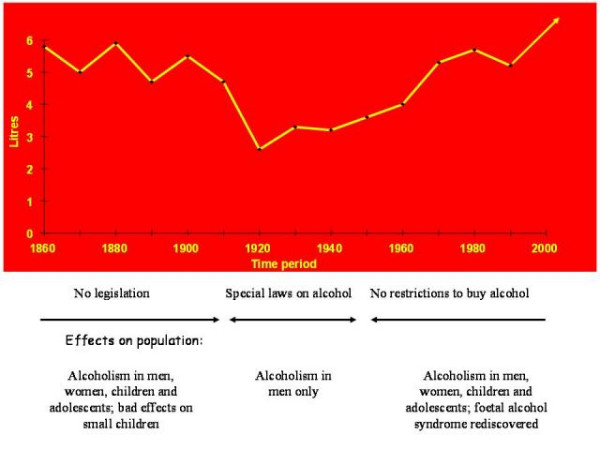
**Swedish alcohol consumption in litres per individual**. Swedish alcohol consumption in litres per individual, > age 15, recalculated as 100% pure alcohol. Five-year average. Health effects described in the population.

Until 1977, there was a special National Alcohol Register on alcohol related criminality including public drunkenness. Using this register, as shown in Table 1, it was possible to describe the relationships between criminality and alcohol abuse in previous prospective cohort studies of CAP patients, average children and youth and risk groups. It is a disadvantage that such an analysis could not be run for the Jämtland cohort of today. However as the previous Jämtland CAP patients registered for criminality needed general psychiatric care as adults due to diagnoses of substance-related disorders and personality disorders, it seems relevant to assume that the rise in the consumption of alcohol in the Swedish population is one of the factors explaining the current findings of higher criminality in boys and girls from the Jämtland cohort compared to the Stockholm cohort from the 1950's (Figure 1). The relevance of this assumption, and the effect on criminality of the changes in the Swedish consumption of alcohol from 1916–1977 when Sweden had special Temperance Laws and until today, is supported by the results from Otterström in 1946 [19] and the results from Rydelius [4] and Rydelius & Nylander [20].

In Otterström's study, [19] she followed 2346 of the children who came into contact with the Child Welfare board of Malmö, Sweden, between 1903 and 1940, four to thirty one years after their referral. She made a separate study of children of alcoholic fathers (she also commented upon the fact that "Mothers are not taken into account as the number of women who are drunkards is relatively small"). She found that "In summing up it appears that whether the whole material or the different groups are examined separately the same conditions are found, namely that the sons run a considerably greater risk of being convicted of a serious crime if the fathers were addicted to alcohol". She found no such relationships for girls to alcoholic fathers. However she did put forward the following question: "It may be asked whether the children of drunkards themselves become addicted to alcohol when they grow up" and compared sons of alcoholic fathers to non-alcoholic fathers. She found only a weak support for this hypothesis: "Calculation of the total risk shows that at the age of 35 the difference between the two categories is almost statistically probable".

In Rydelius' prospective study of "Children of alcoholic fathers" from 1958–1978 [4] it was found that boys of alcoholic fathers more often than boys of control fathers developed **both **criminality and alcohol addiction (and co morbid use of illegal drugs as well). Concerning boys, similar results were also found by Nylander & Rydelius [20] when comparing "Children of alcoholic fathers from excellent versus poor social conditions" followed from 1961–1967 up to 1981. In these three previous studies similar results were found for girls who showed a low risk of later criminality. This indicates the effects of a secular trend over the past 30 years as more girls were found to be registered for criminality in the present study compared to the CAP patients from the 1950's. This secular trend with an increasing number of girl patients later registered for criminality is in line with Kjelsberg's recent findings in Norway [21].

54% of the boys and 21 % of the girls in this cohort of CAP-patients were registered for criminality at the end of the follow up.

The findings are in line with Scandinavian studies outside Sweden. In Norway, Kjelsberg followed 1,276 former CAP inpatients, ages 15–33, after their stays in the hospital; 1,095 could be traced [22,23]. After excluding those who were entered in criminal registers at the time of their hospitalisation, 932 persons remained – of which 44% appeared in criminal databases. There is no significant difference between the two studied groups.

Concerning boys, the findings are also in line with earlier Swedish longitudinal studies (see table 1). In these studies males were comprehensively investigated, but no in-depth analyses of female criminal trends were made. This study uncovered a rather large percentage of convicted females, and this calls for further analysis.

Results from a 20-year prospective follow-up study of patients treated at the Child Guidance Clinics in Stockholm during 1953, 1954, and 1955 [12] were used to compare risk for later criminality among former Swedish CAP patients.

### An overview of changes of supportive systems for children in Sweden

According to the Swedish statistics on crimes, "since 1950 overall reported criminality has increased dramatically" [24].

Compared to the CAP-patients from the 1950's [12] a similar situation was found in the current cohort, where the criminality risk increased for boys and girls; 23% of the boys from the 1950s entered the Criminal Register on one or more occasions – compared to 54% of the boys in the new cohort. Of special concern is that risk for criminality among girls increased over a 20-year period from 3% among those treated in the 1950s to 19% among those who were treated between 1975 and 1990 and followed over a 20-year period. How should this be explained?

In Sweden, strong supportive systems for children were established between 1920 and 1970. A child social-welfare law was passed in 1924, which forced each municipality to create a planning board for children's social well-being including efforts from the society to focus on a more strict social control to prevent delinquency. The school system was investigated as a means of delivering services. From 1940 to 1970, a differentiated public school system (based on curative education) was established. Two parliamentary decisions (1946 and 1958) established CAP care as a free service to all children, youth, and their families. In- and outpatient clinics and treatment homes had to be established in every county council. Since the 1920s, when CAP care was first launched, school psychiatry was established to support mentally retarded and learning disabled children. An important objective when passing the child social-welfare law and enabling free CAP services was to prevent and reduce juvenile delinquency.

Until the 1970s, treatment and support given to children, youth, and parents were based on close co-operation among CAP units, mental health staff at schools, and child social-welfare boards in the municipalities. Multidisciplinary treatment strategies included combinations of medical methods; individual psychological treatment of children; educational methods, "change" at schools; parental treatment; and environmental change (treatment home, foster home, and adoption).

From the 1970s until today, a great change occurred in the then available support systems for children. Starting in 1969, the public school system, based on principles of curative education, reorganised. Teaching strategies based on the individual's cognitive capacity in small groups was replaced by an "inclusive" school in which special education and small groups were looked upon as "excluding" factors and thus abandoned. Mental health staffs in the public schools were minimised. In many places, positions such as school psychiatrists, social workers, and school psychologists were eliminated and just a few school nurses remained. From 1982, the child social-welfare law changed. The system with specialised child welfare boards in each municipality was abandoned in favour of generalised boards that deal with overall social services for inhabitants. Generalists replaced child social-welfare workers who were especially trained to work with children and youth. In many sites in Sweden, treatment strategies, based on theories from psychodynamics and family theories, replaced child psychiatric multidisciplinary approaches that were used until the 1970s. Consequently, close co-operation among CAP units, school psychiatry, and child social-welfare units declined.

The findings may be a key for understanding risk for criminal development in CAP clinical patients. It is unlikely that psychiatric care causes criminality, but it has not prevented it. Taken together, these factors probably contributed to current higher criminality risk among CAP patients:

• Rise in alcohol consumption in the Swedish population from the mid-1950s (when alcohol restrictions stopped; see Figure 1),

• Changes in the organisation of child social welfare work and in the school system,

• Treatment strategy changes in Sweden's CAP care,

• Declining (starting in 1970) co-operation between CAP, school psychiatry, and child social-welfare.

### Possible mechanisms to understand future delinquency

Patients admitted to CAP care are a more vulnerable group compared to average children and youth, why their risk for future criminality might be increased. In their study of 111 young Swedish male criminals (examined by a forensic psychiatrist), Adler et al [25] found a multifactorial background that involves constitutional vulnerability together with social problems and school failures. The most important risk factors associated with juvenile criminality were malfunctioning parental care and supervision, school problems, influence from criminal companions, unstable family situations, and unemployment, suspension from school, and heavy alcohol and narcotics use.

In this study, malfunctioning parental care and supervision, behaviour disorders and school problems were obvious risk factors. Those, who later developed criminal behaviour, also received more in-ward psychiatric care than other CAP patients.

According to Hirchis theory [26] about social bonds, the relevant question is not what makes certain individuals commit crimes but rather what prevents us all from committing crimes. The answer rests with social control, which is implied through integration into social systems i.e. into the family, school and the surrounding society.

In this study, split family was one of the variables that had an effect on criminal development. In a split family, there is an increased risk for deviant behaviour and criminality among the children [27]. A basic theory about the connection between a split family and criminal behaviour of children is what Loeber and Stouthamer-Loeber calls the "splitting paradigm" theory [28].

Split family may in turn be the result from parental malfunctioning leading to failure of social control in the families. Rutter [29] states that it is crucial to differentiate between risk indicators and risk mechanisms. For example, a family split-up doesn't necessarily put the child at risk, but the process that arises from the split-up creates risk mechanisms such as single-parent problems or parent conflicts in which the child might be involved and where social control is lacking.

The changes in the Swedish organisations of child social welfare work and in the school system and in the treatment strategies in Sweden's CAP care may perhaps together with the declining co-operation between CAP, school psychiatry, and child and adolescent psychiatry as described above put children at risk in a worse situation today compared to the situation 40 years ago when their need of social support and social control has been neglected. Of special concern in this respect is that group of schizophrenic patients in this study more frequently committed violent crimes.

## Limitations

The population in Jämtland, living in one of Sweden's smallest county councils, may not be representative for the average Swedish population, why the generalisation of the results could be limited. But a comparison (unpublished) between CAP inpatients in Jämtland and in Stockholm in 1981 showed only a few significant differences between the patient groups. Another disadvantage with the method was that it was based on data from psychiatric hospital records, although containing data from parents, patients, child social welfare and schools, they were initially based on ICD 8 and 9 – but re-categorized as per ICD-10.

Although a protocol was set up for the study, it was based on hospital records. As they are not in all aspects reliable scientific sources it is a limitation for the study.

When comparing the Jämtland cohort to the Stockholm cohort from the 1950's it is essential to discuss the following three concerns: the age and sex differences that exist comparing the two cohorts together with the change in the Swedish Registers on registrations on alcohol problems that occurred in 1977.

## Conclusion

Over the past 50 years, the percentage of Swedish boys admitted to CAP care and later registered as criminals seems to have doubled. The percentage of girls increased almost seven times. While individual and family factors seem to be very much the same, comparing results from CAP patients from the 1950s to the current cohort, this higher risk of later criminality is hypothetically the result of rising alcohol consumption in Sweden, co morbid use of illegal drugs, and changes in the organisation of child social welfare work, the school system, and CAP methods that occurred since 1970. In turn this may have put children at risk in a situation where their need of social support and social control has been neglected.

## Competing interests

The author(s) declare that they have no competing interests.

## Authors' contributions

UE collected the data, performed the statistical analysis, and drafted the manuscript. PAR participated in the study's design and drafted the manuscript. Both authors read and approved the final manuscript.

## Pre-publication history

The pre-publication history for this paper can be accessed here:


